# Psychosocial challenges facing women living with HIV during the perinatal period in rural Uganda

**DOI:** 10.1371/journal.pone.0176256

**Published:** 2017-05-01

**Authors:** Scholastic Ashaba, Angela Kaida, Jessica N. Coleman, Bridget F. Burns, Emma Dunkley, Kasey O’Neil, Jasmine Kastner, Naomi Sanyu, Cecilia Akatukwasa, David R. Bangsberg, Lynn T. Matthews, Christina Psaros

**Affiliations:** 1 Department of Psychiatry, Mbarara University of Science and Technology, Mbarara, Uganda; 2 Faculty of Health Sciences, Simon Fraser University, Vancouver, Canada; 3 Behavioral Medicine Program, Department of Psychiatry, Massachusetts General Hospital (MGH), Boston, United States of America; 4 Division of Global Health, Massachusetts General Hospital (MGH), Boston, United States of America; 5 Research Institute, McGill University Health Centre, Montreal, Canada; 6 Oregon Health Sciences University-Portland State University School of Public Health, Portland, OR, United States of America; 7 Division of Infectious Disease, Massachusetts General Hospital (MGH), Boston, United States of America; 8 Harvard Medical School, Boston, MA; Universita Cattolica del Sacro Cuore Sede di Roma, ITALY

## Abstract

The complexities of navigating pregnancy while living with HIV predispose women to additional stress. Finding ways to minimize psychosocial challenges during the perinatal period may maximize the well-being of mothers living with HIV and their children. The goal of this study was to explore psychosocial challenges experienced by women living with HIV (WLWH) during pregnancy and the postpartum.

We conducted individual in-depth interviews with 20 WLWH recruited from an HIV treatment cohort study in Mbarara, Uganda as part of a larger study exploring perinatal depression. We conducted content analyses to identify themes related to challenges of WLWH during pregnancy and the postpartum. Participants had a median age of 33 years [IQR: 28–35], a median of 3 living children [IQR: 2–5], and 95% had achieved HIV-RNA suppression. Challenges were organized around the following themes: HIV -related stigma from health professionals, HIV status disclosure dilemma, unintended pregnancy and intimate partner violence, HIV and environmental structural barriers and distress and fear related to maternal and child health. Stigma centered on discrimination by health care professionals and personal shame associated with being pregnant as a WLWH. This led to difficulty engaging in HIV care, particularly when coupled with structural barriers, such as lack of transportation to clinic. Participants experienced intimate partner violence and lacked support from their partners and family members. Distress and fear about the health and uncertainty about the future of the unborn baby due to maternal deteriorating physical health was common. The perinatal period is a time of stress for WLWH. Challenges experienced by WLWH may compromise successful engagement in HIV care and may reduce quality of life for women and their children. Strategies aimed at alleviating the challenges of WLWH should involve the larger structural environment including partners, family and community member as well as policy makers, funders and program implementers to work together for the common cause. These consolidated efforts may not only lower the risk of psychological distress but has potential to create long lasting solutions to benefit the wider community.

## Background

Globally, HIV disproportionately affects women compared to men [1]. In Uganda, 8% of women are living with HIV, compared to 6% of men [2]. Reasons for women's higher HIV burden relate to both physiological and socio-structural factors [3–6]. In particular, women face gender and power inequity, economic vulnerability and dependence, and gender-based violence, which may limit options for negotiating intimate relationships and safer sex [5, 7–10].

Many of the socio-structural factors that increase women's risk of HIV acquisition [4, 7, 11–18] also compromise linkage and retention in HIV care [19–25]. Such gaps have significant consequences for women living with HIV (WLWH), in terms of health, quality of life, and survival, in addition to the risk of HIV transmission [24, 26–32].

For WLWH who become pregnant, the stakes of engaging in HIV care are amplified, given the risks of perinatal transmission [33, 34]. In Uganda, where Option B+ has been the standard of Prevention of Mother to Child Transmission (PMTCT) programming since 2014 [35], antenatal clinic HIV prevalence is 7% and in 2014, 84% of pregnant WLWH were enrolled in Option B+ and receiving HIV care [36–38]. Uptake of services to prevent perinatal HIV transmission is limited by many factors including limited information, fear of unintended HIV disclosure, lack of support from intimate partners, perceived HIV and pregnancy stigma from the community and health care providers, and women’s concerns about health implications of long-term antiretroviral treatment [39, 40]. As a result, pregnant WLWH are less likely than non-pregnant WLWH to get tested for HIV, access HIV care, and initiate ART [29].

For many WLWH, pregnancy and the postpartum periods introduce additional stressors related to individual partnership dynamics, and community and healthcare expectations [41–44]. Although quantitative evidence suggests that the postpartum period, in particular, is associated with poorer adherence to ART and higher rates of disengagement from HIV care [41, 43, 45–47], there is limited understanding of the types of psychosocial challenges facing pregnant and postpartum WLWH, particularly in rural settings, where availability of HIV and mental health services may be more limited. Thus, the goal of this study was to explore psychosocial challenges experienced by WLWH living in rural Uganda during pregnancy and the postpartum.

## Materials and methods

### Study setting

We conducted this study in Mbarara a rural town in Southwestern Uganda located approximately 270km from Kampala, the capital city. Mbarara town has an estimated population of 195,013 people [48]. Adult HIV prevalence in the region is estimated at 8%, and women carry a higher burden of infection with a reported prevalence of 9% compared to 7% in men [49, 50]. All study participants were accessing care at a public HIV clinic within the regional referral hospital. The clinic offers comprehensive HIV care, including ART, free of charge to patients.

### Study participants and recruitment

Study participants were WLWH recruited from the Uganda AIDS Rural Treatment Outcomes (UARTO) cohort study [51]. From 2005–2015, UARTO followed over 700 adults (≥ 18 years of age) men and women living with HIV who initiated ART at study enrolment, and who were receiving care at the local HIV clinic, and living within 60 km of the clinic site. Data from these analyses comes from a qualitative sub-study of the experiences of depression among WLWH during pregnancy and the postpartum period. Eligible participants for this sub-study were females, enrolled in the parent UARTO cohort study, and had experienced a pregnancy in the last two years prior to recruitment. The primary aim was to explore experiences of depression among WLWH during pregnancy and postpartum. We used purposive sampling to select eligible participants with a range of experiences based on their responses to the Hopkins Symptoms Checklist (HSCL-16) within the parent cohort study.

The primary research objective was to explore women’s experiences with depression during and after pregnancy. Because women were recruited from a cohort study where depressive symptoms were surveyed quarterly, we took advantage of these data to capture a range of women’s experiences with depressive symptoms. Cohort participants were screened for depression symptom severity using a modified version of the HSCL-15 for depression. Based on previous studies using HSCL in Uganda, a 16th item was included, “Feeling like I don't care about my health” [52]. Each of the 16 symptoms is scored on a 4-item Likert scale ranging from not at all (1), a little (2), quite a bit (3), to extremely (4), and the total depression severity score was calculated as the mean of the 16 items, with higher scores indicating greater depression symptom severity. We considered a dichotomous measure of “probable depression”, defined as an HSCL-16 score > 1.75, which has been previously used as a positive screen for depression [52–57]. We recruited 4 groups of women–those with stably high scores (N = 3), those with stably low scores (N = 7), and those with rising (N = 6) and those with falling scores (N = 4), all during pregnancy and postpartum.

A trained research assistant contacted potential participants by phone and explained the purpose of the study, the anticipated benefits, and the risks of participating. If interested, the research assistant scheduled an interview at a location and time chosen by the participant. The informed consent process took place on the interview day, ensuring voluntary participation, confidentiality and safety. Participants received transport reimbursement of approximately 5 USD. As noted above, the analyses presented here are related to an emergent theme of psychosocial challenges experienced by the participants.

### Data collection

We conducted semi-structured interviews with twenty participants from February through August 2014. We conducted one on one in depth interviews with participants according to guidelines outlined by Pope and Mays [58].The questions were designed to capture experiences of WLWH during the perinatal period, and were developed through input from mental health care providers and experts in reproductive health, HIV, and safer conception practices. The interview guide was piloted extensively among the study staff to assess its clarity and content, and revised accordingly by removing some questions and rephrasing others. Interviews lasted approximately one hour. The interview guide (S1 File) included questions about WLWH’s experiences during pregnancy and the postpartum, women’s thoughts and feelings about becoming pregnant, how their HIV status influenced their thoughts and feelings towards pregnancy, and their partner’s thoughts towards the referent pregnancy. The final section of the interview explored feelings and experiences of the participants following childbirth. Ugandan research assistants trained in qualitative research methods and fluent in English and the local language (Runyankore) conducted the interviews and were blinded to participants’ quantitative depression scores and patterns.

### Ethical considerations

All participants provided voluntary written informed consent at study enrollment. The Institutional Review Committee, Mbarara University of Science and Technology; the Partners Human Research Committee, Massachusetts General Hospital; and the Research Ethics Board of Simon Fraser University approved the study. Consistent with national guidelines, we received clearance for conducting our study from the Uganda National Council for Science and Technology and from the Research Secretariat in the Office of the President.

Given the focus of the study, we developed and implemented a protocol for referring distressed participants to a psychiatrist at the mental health clinic at the recruitment site. At the beginning of the study, a Ugandan psychiatrist (SA) trained all research assistants to recognize signs and symptoms of depression. If the research assistants noticed signs or symptoms of acute and severe distress, they were instructed to refer the participant to the mental health clinic. If the research assistants noticed that the participant needed counseling related to HIV care, the research assistant referred the participant to the counselor in the HIV clinic. Two participants (one with symptoms of distress and another in need of counseling on HIV care) used our referral protocol over the course of the qualitative sub-study.

### Data analysis

Demographic information for each participant was collected from the UARTO cohort database. We conducted one-on-one in depth interviews with participants according to guidelines outlined by Pope and Mays [58].Research assistants translated and transcribed audio recordings of the interviews into English. Transcripts were reviewed by research assistants and a psychiatrist (SA) to assess translation quality and fidelity. NVivo 10 (QRS International) was used to facilitate analyses. Content analysis was used to conduct initial analysis of the data, exploring the experiences of depression among WLWH during pregnancy and the postpartum, according to Strauss and Corbin [59]. Transcripts were read by 9 research team members to identify major themes and to inform development of a coding scheme to categorize the data. The final coding scheme included both a priori themes and those which emerged from preliminary readings of the transcripts. The codebook guided the coding process, which was completed by two members of the research team (BB and ED). The two coders compared coding for four interviews to ensure coding reliability and to verify understanding of the codebook (Kappa statistic = 0.82) [60] and then coded the remaining interviews independently. After coding all interviews, the research team further discussed the emergent themes in the context of coding.

Themes relating to psychosocial challenges experienced by WLWH emerged from the data as an independent theme, and were explored through an iterative process using techniques described by Miles and Huberman [61]. Data were further organized into themes and sub themes relating to psychosocial challenges faced by WLWH during pregnancy and the postpartum [59]. Data reduction methods were employed to extract the overarching narrative from the most pertinent data.

## Results

Forty-two (42) participants were eligible to participate in the study and 20 participants were recruited. We aimed to recruit a similar number of participants in each category, however only 5 of 42 eligible participants had steadily high HSCL scores. Participants had a median age of 33 years (range 22 to 40) and 95% were virally suppressed at the UARTO visit closest to the interview date. Women had a median of 3 living children and a median CD4 cell count of 677 cells/mm^3.^ (Table 1). Of the 20 women, 18 (90%) had a live birth and 2 (10%) experienced other pregnancy outcomes.

**Table 1:** Socio-demographic characteristics of women living with HIV who had a pregnancy within 2 years prior to interview (n = 20)

Characteristic	Median (IQR)
Age (years)	33 (28, 35)
Time on ART (years)	2.3 (1.8, 5.1)
Most recent CD4 cell count (cells/mm^3^)	677 (440, 767)
Number of live births	4 (2, 6)
Number of living children	3 (2, 4.3)
Number of biological children	2 (1.5, 3)
Time between pregnancy outcome and interview (months)	15 (7, 21)

### Challenges

Data from 20 interviews was summarized into 5 themes that emerged as major challenges faced by WLWH during pregnancy and the postpartum: (1) HIV-related stigma from health professionals, (2) HIV status disclosure dilemma, (3) unintended pregnancy and intimate partner violence, (4) HIV and environmental structural barriers, (5) distress and fear related to maternal and child health. Each of these challenges is discussed in detail below:

#### HIV -related stigma from health professionals

Participants shared their experiences about HIV-related stigma during pregnancy and the postpartum which centered on shame associated with having children as a WLWH. Participants experienced stigma and discrimination whenever they went to hospital for antenatal care services. WLWH were treated negatively by health care providers compared to HIV positive women. In addition, WLWH received negative messages about being pregnant while HIV-infected from health workers to discourage them from reproducing. As a result of the negative information and reception from health care professionals as WLWH sought care some women expressed personal shame around being HIV-infected and pregnant:

Sometimes when you get to the [health] facility and they look at your documents and they realize you are infected [HIV+], they do not handle you [as] well as that one who is not infected.*—*(34 year-old woman, 7 years living with HIV)

It was as if it was a taboo. To be sick [HIV positive} and then you get pregnant. I was saying “if I come here [HIV clinic] to get medicine and they see me pregnant, will they not call me a fool?” “You have HIV and you are pregnant?”–(40 year old woman, 8 years living with HIV)

Some women received unexpected reactions from health workers after learning of their pregnancy. Instead of support, women received criticism and blame from health workers for becoming pregnant and were openly discouraged from reproducing because of their HIV status:

She [health worker] didn’t help me … because she was like, “how can you get pregnant? Mmh, now you think you get pregnant without telling us. You are supposed to ask us instead of telling me that it has already happened”… I tried explaining to her but she kept laughing, I just kept quiet.—(31 year-old woman, 9 years living with HIV).

#### HIV status disclosure dilemma

Although participants had a strong sense of obligation to disclose their HIV status they feared the negative reactions of disclosure, including fear of abandonment and intimate partner violence. Women worried that disclosing their positive HIV status to their partners would lead to assumptions that they had been unfaithful to the partnership and they feared the emotional and physical violence that would follow. Despite this dilemma some women were brave enough to disclose although deep inside they expected the worst reaction from their partners:

I started thinking, will I deliver this baby alive, and won’t the man himself kill me? In fact I even first hid the papers [HIV test results] and kept quiet. But later my heart told me, tell him. If he leaves me, he leaves me, so long as he takes care of the pregnancy. He instead told me let’s go to hospital … and they tested us and told us that the man is negative but I was positive [HIV positive]. I told him, leave me alone, you are negative, I am positive, [and] how are we going to live together?… He said, it’s not possible [to leave me], I kept quiet and we stayed together.—(35 year-old woman, 3 years living with HIV)

In addition to the fear and distress about impending partner violence and abandonment following disclosure women also anticipated negative reactions associated with HIV within the community, including gossip and discrimination. To avoid this, some women kept their HIV serostatus a secret:

What happens at our village, you find people talking about you [HIV positive women], so and so is sick [HIV positive], but does a sick person look like this…. You see someone trying to ask you and know about your status but you keep quiet because when you tell them they spread it in the community.–(28 year-old woman, 6 years living with HIV]

#### Unintended pregnancy and Intimate partner violence

Some participants reported experiences of violence, particularly after informing their partners of a pregnancy. Verbal and emotional abuse by partners was common especially when women conceived without the knowledge of their partners. Women’s intentions and intelligence were questioned by their partners because of their status and pregnancy. Some participants were denied access to treatment by their partners which interrupted their treatment adherence and others were denied a chance to work in order to provide for themselves during pregnancy and the postpartum:

When I told my partner that I was pregnant for the second time, it did not amuse him. He did not take it well, because he abused me, (saying) “by the time you got pregnant were you stupid?”—(34 year-old woman 7 years living with HIV)

After giving birth he started treating me badly he knew that he had finished me off because he had already infected me with HIV. I no longer had anywhere to go. He stopped me from working and stopped me from coming to hospital to pick my medications [ARVs.—(26 year-old woman 6 years living with HIV)

#### HIV and environmental structural barriers

Structural barriers related to HIV and the environment complicated women’s perinatal experience. Participants endured financial constraints and struggled to sustain themselves and the baby. The stress of balancing work, pregnancy and childcare as an HIV positive woman was hard to endure. Some of the women spent more time at work which interfered with the care their children received. Financial constraints limited the women’s access to HIV care due to lack of transport fare to the HIV clinic compromising their physical health. Women described these situations as being stressful:

You see that stress never stopped there [HIV and pregnancy], I failed to stay with her [child] because of work. I had her [child] for two months before I went back to work, the third month I was fidgeting with her but I saw that she [child] needed to be attended to. She would be in the house [where the woman worked] crying, and everyone would be concerned. I gave her [child] to my friend so I could continue working.–(36 year-old woman, 7 years living with HIV)

At times we [WLWH] meet financial problems. For example when you are supposed to come and get more drugs [ARVs] you find that you do not have money for transport. You go to a friend to borrow money and you find that she does not have it. So you exceed your appointment dates for a week or more. And when you come to collect your drugs the doctors do not treat you well. That also makes us feel bad.–(31 year old woman, 7 years living with HIV)

Additionally, women experienced challenges associated with dependence on their partners to satisfy basic needs, while navigating polygamy and multiple partnerships. Some participants reported their partners to be involved with other partners, which complicated their relationships. In addition to interfering with the stability of relationships, polygamy and multiple partnerships compromised the financial support women received from their partners which in turn affected their physical health:

There is no good relationship with my partner because he has a second wife. He does not look after me properly; all the money goes to the other side [second home]. He does not bring for me the good things [food] that I need to eat to sustain my health.*—*(36 year-old woman, 8 years living with HIV)

Some participants struggled with cultural norms that deny women reproductive autonomy, which prevented them from making decisions concerning having children and utilizing available contraception methods. Some women reported that pregnancy resulted from their partners and partners’ families wanting children, rather than their own desire to have children. Even when women did not want to conceive, they had to comply with and fulfil their partners’ desires:

After the first girl [child] I started family planning, the injection for three months. But eventually I stopped because my mother in-law and father-in-law were accusing me that I gave birth to one child like an antelope.–(26 year-old woman, 6 years living with HIV)

#### Significant distress and fear related maternal and child health

The experience of pregnancy while living with HIV resulted in significant anxiety across several domains. Participants were distressed about the possibility of HIV transmission to their unborn babies, and feared that their babies would die soon after birth due HIV:

I kept saying even if I deliver him, will he live? Maybe he will fall sick from the womb because of my poor strength. I would think, “Now I don’t have enough care, life is deteriorating, won’t I deliver the baby and he just dies”.*—*(36 year-old woman, 10 years living with HIV)

The distress and fear about the babies’ health were further fuelled by the women’s own deteriorating health during pregnancy as women feared they would not be strong enough to work and provide for themselves and their children, or that their children may end up as orphans:

I used to think that I am going to have this child, and fail to get what [resources] to take care of him … and he will eventually die. I thought I would have no strength to educate him because I would be helpless and not be able to work for myself because of HIV. I was also thinking that if I fall sick I will no longer be strong enough to work for him, get food to feed him, and he would eventually die.*–*(28 year-old woman, 3 years living with HIV)

The fear that their children would end up as orphans brought on many questions concerning the status of the children and how they would adhere to the medications without their mothers. Participants worried had distressing thoughts about how they take care of an HIV positive child knowing he would die but also worried about who would give appropriate care to the children if the mother dies. This distress was persistent throughout the infant testing period:

I was so scared that, now my baby is sick and if they tell me that he is sick, what will I do? I kept thinking, now will I look after him; put him in school knowing that he will die. Now what if I die and am buried who will ever look after him? But even in the village at his grandmother’s place how will he end up there? He is already on medicine [ARVs], who will bring him [to the HIV clinic] to get medicine? I have not disclosed to my in-laws, now if I die without disclosing that the child is sick, who will pick his medicine [ARVs], who will take him there [HIV clinic]?. I will die today and he [child] dies tomorrow.-(36 year old woman, 3 years living with HIV)

Women struggled with distressing thoughts about their young children taking ARVs in case they were perinatally infected with HIV. The thoughts centered on the ability of the children to tolerate the side effects of medications and the effects of the medications on children’s growth and development:

I was so bothered and suffering that I am going to have a child that is HIV positive.”What was I going to do?”“Finding me at the pharmacy collecting drugs for myself and then for my child?”—(26 year old woman, 6 years living with HIV)

P: I felt bad, I was thinking; and I was like I am sick [HIV positive], then the child is also sick [HIV positive]…… the thoughts were many.

I: What were you thinking about exactly?

P: Thinking about my child and how long she will take these tablets [ARVS]. When and how will she grow when she is on these tablets [ARVs]? At least for me I am old and can handle [side effects of ARVs]. But to start drugs when she is a baby?…eeh that was so disturbing.*–*(22 year old woman, 2 years living with HIV]

## Discussion

Participants in this study described a myriad of challenges during pregnancy and the postpartum, including experienced stigma, discrimination, and criticism from health workers, community members, and violence from partners associated with being pregnant as a WLWH. While some of the participants were openly criticized by healthcare professionals for being pregnant, others were worried about the reception at the health facility based on a personal belief that being pregnant while living with HIV was shameful. Women reported structural challenges, including poverty and cultural norms that uphold men as the decision makers, as factors that worsened HIV-related challenges during pregnancy. Physical violence and emotional and sexual abuse from intimate partners were common among study participants. Women also experienced stress, anxiety and worry related to their own health, the health and future of their babies, and the risk of HIV transmission. Understanding these challenges can inform interventions to support WLWH to better navigate HIV care during pregnancy and postpartum, adhere to PMTCT recommendations, and support their psychosocial needs to improve mental and physical health outcomes.

Our study findings parallel with findings from a previous study in Ethiopia whereby WLWH were threatened with withdrawal of their HIV care services if they became pregnant [62]. Similar to our finding, reports of verbal abuse, neglect by health care workers, and social isolation within communities against pregnant WLWH have been documented [4]. As a result, some WLWH drop out of HIV care due to the stigma associated with HIV, while others attempt to conceal their HIV status for fear of stigma, discrimination, and negative judgment within the community [47, 63–65]. HIV-related stigma prevents many pregnant WLWH from initiating and adhering to antiretroviral therapy [66–68]. In order to realize positive benefits from the implementation of HIV prevention and treatment strategies including PMTCT programs, efforts to reduce HIV-related stigma in pregnancy must be prioritized [69].

Disclosure of HIV status was a major challenge among our study participants due to anticipated and real negative consequences especially from intimate partners. Our findings are in agreement with previous studies that have reported intimate partner violence, blame, and abandonment associated with disclosure of an HIV positive status among WLWH [27, 70]. As a result, many women refuse to test for HIV to avoid disclosure, [71, 72] which limits their ability to engage and adhere to PMTCT care [47, 66, 69, 73, 74].

Other findings indicate that women experienced intimate partner violence in the form verbal, physical, emotional and sexual abuse. This is consistent with findings of previous studies that intimate partner violence is commonly perpetrated against WLWH [75–77]. In a study in South Africa, WLWH who conceived without discussing their pregnancy intentions with their partners experienced more violence from their partners than WLWH who did discuss their pregnancy intentions [78]. Violence against pregnant WLWH compromises physical and emotional well-being and interferes with engagement in HIV care and adherence to medications, hence increasing the risk of perinatal HIV transmission [79–82]. In addition to coping with HIV-related stigma, HIV status disclosure, and intimate partner violence, these challenges were amplified by poverty and cultural norms, which put women in a vulnerable position. [8, 9, 16, 83]. Poverty and economic hardships often force WLWH to endure abusive or unhealthy relationships to retain economic support from their partner [84].

Women in this study reported experiencing distress, fear and worry about their own health and the health of their babies and about being unable to provide for themselves and their children due to ill health. Anxiety and distress associated with HIV among women has been reported in previous studies [85–87]. The experience of pregnancy and postpartum among WLWH has been characterized by negative emotions including fear, anxiety, guilt, and sadness in previous research [85, 86]. In our study, we found that fear and distress among WLWH during the perinatal period centered around the health and status of the baby persisting throughout the infant HIV testing period, which is consistent with findings from previous research [88, 89].

Our qualitative study had some limitations. The study had a small sample size, therefore the views of the participants may not represent the challenges of all WLWH during pregnancy and the postpartum. We interviewed women who were enrolled in HIV care and part of a cohort study where they received regular reviews in the HIV clinic and other kinds of support, including transport reimbursement and other incentives like cooking oil. This could have resulted in reporting bias. Because most of these women were enrolled in care for over five years, their experiences may be different from those women who are early in the HIV and PMTCT treatment cascade.

## Conclusions

Efforts to eliminate perinatal transmission may not be successful until the psychosocial challenges experienced by WLWH along the treatment cascade are addressed. Women continue to experience stigma and discrimination, fear of disclosure, gender inequality, intimate partner violence, and economic hardship. These challenges prevent women from testing for HIV and also impair their ability to adhere to medications, which may compromise their physical health and the health of their children.

To enable WLWH to successfully navigate HIV care during pregnancy and the postpartum, strategies aimed at encouraging HIV testing, safe disclosure, and involving male partners in antenatal care should be considered [24]. Creating opportunities to address intimate partner violence are likely to decrease the stress and negative consequences of disclosure, and may enable many women to access HIV care services [14]. Efforts to promote gender equity and economically empower women will enable WLWH to make autonomous decisions concerning their health, engage in care, and adhere to PMTCT treatment recommendations during pregnancy and the postpartum period [16].To help WLWH overcome the challenges that they grapple with, community members, partners, health care providers and family members and larger structural environment including policy makers, funders and program implementers should be involved and work together to empower communities in general through which WLWH can benefit [90]. Previous research has documented that partner and family and community support is instrumental in alleviating challenges faced by WLWH lowering the risk of psychological distress in this population [90, 91].

Interventions that seek to mitigate the effects of these stressors on WLWH, particularly during the perinatal period, may maximize the well-being of women and their children. The psychosocial challenges that impair women’s ability to engage in HIV care and adhere to ARVs should be explored and subsequently addressed at community level to enable WLWH to navigate the HIV treatment cascade.

## Supporting information

S1 File Interview Guide Postpartum and antenatal depression among women enrolled in Uganda AIDS Rural Treatment Outcomes Study (UARTO) (DOCX)
