# Supplementary material for: Psychosocial challenges facing women living with HIV during the perinatal period in rural Uganda
Source: PLoS One. 2017 May 1;12(5):e0176256. doi: 10.1371/journal.pone.0176256 (PMC5411062; doi:10.1371/journal.pone.0176256)
Supplement: S1 File — (DOCX) [file pone.0176256.s001.docx]

**Postpartum and antenatal depression among women enrolled in UARTO**

**(HIV-infected women with recent pregnancy)**

UARTO Patient Participant Interview Guide

Hello. My name is _________________________ and I am part of the UARTO team doing a study looking at what problems affect a woman’s mind and emotional health during and after pregnancy. For some people, big life changes can be very difficult, and some women undergo changes in their thoughts, feelings or body. I want to hear about your emotional experiences during and after pregnancy. Our goal is to use this information to help HIV-infected women have healthy pregnancies. I have a series of questions but am particularly interested in your insights, so anything you have to say will be helpful. This interview is informal – like a conversation – and everything you tell me is confidential. There are no right or wrong answers – we just want to hear about your own experiences.

As we discussed, our interview will be audio recorded. This way, we can speak freely and I will not forget the things you tell me. No one other than the UARTO team will be able to listen to the recording, and it will be deleted when we have finished the study.

We have just reviewed the consent form, which describes the study in detail and gives us permission to speak with you. As a reminder, you are not required to answer the questions and may skip any questions that you do not want to answer.

Do you have any questions before we begin the interview?

*Interviewers, please start with the* next *section. Use this first section for women who hesitate to discuss their own experiences.*

(RA warm-up item discussed by Schola and team)

| 1. Tell me about what pregnancy is like for women living with HIV.  (*Interviewers: This is a question about women in general. If participant moves straight to her own experiences, that is fine. You will then skip these items and move to the next section that begins with question #4.)* | *Explore*  What are some reasons women living with HIV may want to get pregnant?  How do women living with HIV feel emotionally during pregnancy?  What are some positive experiences women living with HIV may have during pregnancy?  What are some negative experiences women living with HIV may have during pregnancy? |
| --- | --- |
| 2. What are some emotional or mental health problems that may affect women living with HIV **during pregnancy**? By mental health problems, I am talking about things like feeling nervous or worried or sad. | *Explore*  *If relevant to depression, probe further for emotional & somatic symptoms.*  *Symptoms of depression may include fatigue, sadness, memory issues or trouble concentrating or making decisions, loss of interest in things, changes in sleep patterns, feeling suicidal, feeling unhappy or miserable, crying easily, feeling scared, panicky, worried, anxious or guilty* |
| 2a. For each problem:  Can you tell me more about this?  How does this affect women’s health?  How does this affect daily life?  What do people call this problem?  How do women deal with this problem?  Tell me about how HIV impacts this problem. |  |
| 3. What are some emotional or mental health problems that may affect women living with HIV **in the first few weeks to months *after* pregnancy**? By mental health problems, I am talking about things like feeling nervous or worried or sad. | *Explore* feelings (both positive and negative) and experiences  *If relevant to depression, probe further for emotional & somatic symptoms.*  *Symptoms of depression may include fatigue, sadness, memory issues or trouble concentrating or making decisions, loss of interest in things, changes in sleep patterns, feeling suicidal, feeling unhappy or miserable, crying easily, feeling scared, panicky, worried, anxious or guilty* |
| 3a For each problem:  Can you tell me more about this?  How does this affect women’s health?  How does this affect daily life?  What do people call this problem?  How do women try to fix this problem?  Tell me about how HIV impacts this problem. |  |

Now, I would like to ask you some questions about your own experiences. Specifically, I would like to hear about your most recent pregnancy. *Interviewers, if she has already told you about her own experiences above, please do not repeat the questions.*

| **Topics and Main Questions** | **Probes** |
| --- | --- |
| 4. Please think back to before you got pregnant - what were your thoughts about getting pregnant at that time? | *Explore* plans for pregnancy, desires for pregnancy, expectations |
| 5. How do you think your partner felt about your getting pregnant? | *Explore* her perceptions of his plans for pregnancy, desires for pregnancy and expectations  *Explore whether partner knew her HIV status around the time she got pregnant and how HIV may have affected his thinking. Explore partner’s HIV status.* |
| 6. After you discovered you were pregnant, how did you feel about the pregnancy? | How did your thoughts and feelings about the pregnancy change over time? |
| 7. How did you feel during your most recent pregnancy? I am interested in things that affected your mind. | *Explore* feelings (both positive and negative) and experiences  *If relevant to depression, probe further for emotional & somatic symptoms.*  *Symptoms of depression may include fatigue, sadness, memory issues or trouble concentrating or making decisions, loss of interest in things, changes in sleep patterns, feeling suicidal, feeling unhappy or miserable, crying easily, feeling scared, panicky, worried, anxious or guilty*  What were some of the challenges you faced…  For each feeling (if needed):   - Can you tell me more about this? - Why do you think you felt this way? - How did this affect your daily life? - *If symptoms were problematic,* How did you try to fix or get help for this problem? - How was this different from your other pregnancies? |

Now I’m going to ask questions about the first six months after your most recent pregnancy. Again, I am interested in your emotional experiences and thoughts or things that affected your mind.

| 8. How did you feel in the first weeks and months after your most pregnancy? | *Explore* feelings (both positive and negative) and experiences. (e.g. Tell me your positive experiences in the weeks to months after your pregnancy. What were your biggest concerns in the weeks to months after your pregnancy?)  *If relevant to depression, probe further for emotional & somatic symptoms.*  *Symptoms of depression may include fatigue, sadness, memory issues or trouble concentrating or making decisions, loss of interest in things, changes in sleep patterns, feeling suicidal, feeling unhappy or miserable, crying easily, feeling scared, panicky, worried, anxious or guilty*  For each feeling (if needed):   - Can you tell me more about this? - Why do you think you felt this way? - How did this affect your daily life? - *If symptoms were problematic,* How did you try to fix or get help for this problem? - How was this different from your experience with other pregnancies? |
| --- | --- |
| 9. *[For women who have had a livebirth. Skip this question for women with other pregnancy outcomes.]* Tell me a little about your child and how he/she is doing. | Has your child been tested for HIV?  *If yes,* Can you tell me a little about that?  How did you feel about going for testing?  What was the result?  How did it feel to learn this result?  Did you tell anyone else about this result?  *If not, explore why not.* |
| 10. How did having this [pregnancy outcome] affect your life? | Explore at personal level, family level and perceptions of community.  Explore for positive changes and experiences and negative experiences as well |

Thank you so much for sharing this information about your life, I know many of these questions are personal, but everything you share is confidential.

So I understand… (*Summarize major points of what she has said*).

As a reminder, we’re interested in learning about the changes in women’s thoughts, emotions, and body that occur during and after pregnancy. We are almost done with our interview. Before we finish, is there anything else you believe is important for me to know?

*Wait for response, then finish*: Thank you for your time and honesty. Your participation is a big help to us.
